# HU-Lacking Mutants of *Salmonella enterica* Enteritidis Are Highly Attenuated and Can Induce Protection in Murine Model of Infection

**DOI:** 10.3389/fmicb.2018.01780

**Published:** 2018-08-22

**Authors:** Guilherme P. Milanez, Catierine H. Werle, Mariene R. Amorim, Rafael A. Ribeiro, Luiz H. S. Tibo, Maria Cristina Roque-Barreira, Aline F. Oliveira, Marcelo Brocchi

**Affiliations:** ^1^Department of Genetics, Evolution, Microbiology and Immunology, Institute of Biology, University of Campinas, Campinas, Brazil; ^2^Department of Cellular and Molecular Biology, Faculdade de Medicina de Ribeirão Preto, University of São Paulo, São Paulo, Brazil

**Keywords:** non-typhoidal *Salmonella*, *Salmonella enterica* Enteritidis, live-attenuated strains, nucleoid-associated proteins, HU protein

## Abstract

*Salmonella enterica* infection is a major public health concern worldwide, particularly when associated with other medical conditions. The serovars Typhimurium and Enteritidis are frequently associated with an invasive illness that primarily affects immunocompromised adults and children with HIV, malaria, or malnutrition. These serovars can also cause infections in a variety of animal hosts, and they are the most common isolates in poultry materials. Here, we described *S.* Enteritidis mutants, where *hupA* and *hupB* genes were deleted, and evaluated their potential use as live-attenuated vaccine candidates. *In vitro*, the mutants behaved like *S.* Typhimurium described previously, but there were some particularities in macrophage invasion and survival experiments. The virulence and immunogenicity of the mutant lacking both *hupA* and *hupB* (PT4Δ*hupAB*) were evaluated in a BALB/c mice model. This mutant was highly attenuated and could, therefore, be administrated at doses higher than 10^9^ CFU/treatment, which was sufficient to protect all treated mice challenged with the wild-type parental strain with a single dose. Additionally, the PT4Δ*hupAB* strain induced production of specific IgG and IgA antibodies against *Salmonella* and TH1-related cytokines (IFN-γ and TNF-α), indicating that this strain can induce systemic and mucosal protection in the murine model. Additional studies are needed to better understand the mechanisms that lead to attenuation of the double-mutant PT4Δ*hupAB* and to elucidate the immune response induced by immunization using this strain. However, our data allow us to state that *hupAB* mutants could be potential candidates to be explore as live-attenuated vaccines.

## Introduction

*Salmonella enterica* is usually associated with self-limiting gastrointestinal diseases in high-income countries and is also responsible for a huge global disease burden through two invasive illnesses, *viz*., enteric fever and invasive non-typhoidal *Salmonella* (iNTS) disease (Feasey et al., 2012; MacLennan and Levine, 2013). The non-host-adapted serovars Enteritidis (*S*. Enteritidis) and Typhimurium (*S.* Typhimurium) are generally accepted as the most frequently isolated serovars associated with iNTS. These serovars primarily affect immunocompromised adults with HIV and children with HIV, malaria, or malnutrition (Kotloff et al., 2013; Feasey et al., 2015). In addition to infecting humans, these serovars can cause infections in a variety of animal hosts, and they are the most common isolates in chickens (Foley et al., 2011). In particular, in Brazil, since 1993, *S.* Enteritidis has been the main serovar isolated from poultry materials; it is responsible for human infections and is continually leading to great losses in the national poultry industry (Campioni et al., 2014).

As reviewed previously (Tennant and Levine, 2015), iNTS is a significant cause of mortality and morbidity worldwide. Hence, live-attenuated vaccines are an attractive vaccine platform as these vaccines can confer long-lasting protection to prevent invasive *Salmonella* serovars. Moreover, these vaccines are inexpensive and easy-to-implement (Erova et al., 2016). Vaccination protocols for this platform have been tested in both humans (Maclennan et al., 2014) and animals (Haneda et al., 2011; Desin et al., 2013). To be effective as a vaccine strain, *Salmonella* has to achieve a delicate balance between adequate attenuation and safety in addition to maximal immunogenicity (Dunstan et al., 1998; Galen and Curtiss, 2014). Unfortunately, the currently employed models are still limited and unable to adjust this balance effectively (Keestra-Gounder et al., 2015). Therefore, characterizing new mutant strains with potential application as live-attenuated vaccines is urgent.

Researchers have targeted different genes to induce attenuation in different *Salmonella* strains. While some groups have characterized mutation in genes directly involved in virulence (Galán and Curtiss, 1989; Hindle et al., 2002), others have sought attenuation by deleting important global regulators (Curtiss and Kelly, 1987; Coynault et al., 1996; Hormaeche et al., 1996; Allam et al., 2011). Nucleoid-associated proteins (NAPs) are an important group of proteins that are yet underexplored for vaccine strains. These proteins are important global regulators of gene expression because they can directly influence the genome architecture (Dillon and Dorman, 2010). Of these proteins, the HU protein, comprised of two subunits HUα and HUβ encoded by genes *hupA* and *hupB*, respectively, can be assembled as a homodimer or heterodimer depending on the growth stage and plays an important role in the regulation of pathogenicity-related genes (Schechter et al., 2003). HU mutants exhibit decreased expression of several virulence-related genes (Mangan et al., 2011). Furthermore, *S.* Typhimurium mutant lacking only *hupA* exhibits attenuated virulence in newly hatched chicks (Turner et al., 1998; Mangan et al., 2011).

Our group developed mutant strains for NAPs to assess the attenuation of the resulting strain in animal models. Such assessment could indicate the possible use of these mutations in designing future live-attenuated vaccine strains. Here, we showed the attenuation of *S.* Enteritidis lacking *hupA* and *hupB* and its ability to induce protection in a mouse model.

## Materials and Methods

### Bacterial Strains

**Table 1** summarizes all the strains used in this study. *S. enterica* strain IOC4647/2004 was used as the background strain to obtain the mutant. This strain belongs to the Enteritidis serovar and is classified as phage type 4, designated as SEnPT4. It was previously isolated from a poultry farm in São Paulo state, and its genome sequence is deposited in GenBank under the accession ID: LTDW00000000 (Milanez et al., 2016).

**Table 1:** Bacterial strains and primers used in this study.

Strain	Genotype
SEnPT4	*S. enterica* Enteritidis PT4 IOC4647/2004
PT4Δ*hupA*	PT4Δ*hupA::cat*
PT4Δ*hupB*	PT4Δ*hupB::cat*
PT4Δ*hupAB*	SEnPT4Δ*hupA::FRT*Δ*hupB::cat*

**Primer**	**Sequence 5′→3′**

*hupA*-F	TAGCAAGCGATAAACACATTGTAAGGATAACTTATGAACAAGGTGTAGGCTGGAGCTGCTTC
*hupA*-R	TTCGATAAAACTGTTCACAGTTATGCGTCTTACTTAACTGCCATATGAATATCCTCCTTAGTTC
*hupB*-F	GGTGCGATATAAATTATAAAGAGGAAGAGAAGAGTGAATAAAGTGTAGGCTGGAGCTGCTTC
*hupB*-R	CTTTGTCACATCCCCCGAGGGGATCACGCTTAGTTTACCGCCATATGAATATCCTCCTTAGTTC
*hupA*DT-F	CGACTGCGAAGAACGTGATA
*hupA*DT-R	AAAGCCGCTGGCAGTAAAC
*hupB*DT-F	TCGTACTTCGAAGGATTCAGG
*hupB*DT-R	GTTGATGCGCCCTTGTACTT

To evaluate the effect of *hupA* and *hupB* on SEnPT4, mutant strains were constructed by deleting these genes individually using a one-step recombination system (Datsenko and Wanner, 2000). This procedure created the single mutants SEnPT4Δ*hupA::cat* and SEnPT4Δ*hupB::cat* (hereafter designated as PT4Δ*hupA* and PT4Δ*hupB*, respectively). **Table 1** describes the primers used to obtain and confirm the mutations. PT4Δ*hupA* was transformed by electroporation (Sambrook and Russell, 2003) using the pCP20 plasmid to create the mutant SEnPT4Δ*hupA::FRT*. Then, the P22HT phage was used to transduce the chloramphenicol resistance cassette from PT4Δ*hupB* to SEnPT4Δ*hupA::FRT* to generate the double mutant SEnPT4Δ*hupA::FRT* Δ*hupB::cat* (hereafter designated as PT4Δ*hupAB*).

All strains were maintained in 15% glycerol at -80°C, and Luria-Bertani (LB) broth and agar (15 g/L) were used for routine culturing. When necessary, chloramphenicol was used at a final concentration of 30 μg/mL.

### *In vitro* Growth

*Salmonella* strains were streaked onto fresh LB agar plates and incubated at 37°C overnight. On the following day, some colonies were re-suspended in LB broth to obtain OD_600_ of 0.4. Next, 1 mL of this suspension was inoculated in 50 mL of fresh, pre-warmed LB broth and incubated with aeration (150 rpm) at 37°C. At hourly intervals from 0 to 12 h, samples were subjected to OD_600_ readings and counting of viable cells on LB agar plates. Counts were reported as the number of colony forming units per mL (CFU/mL). All growth determinations were performed in triplicate.

### Motility Assay

Motility assay was performed to compare the ability of wild-type and mutant strains to move in semi-solid media, as previously described (Sha et al., 2004). In brief, Fresh LB plates containing 0.35% agar were inoculated with 1 μL of cell suspension, prepared by re-suspending cells from overnight LB plates in LB broth, to obtain OD_600_ of 0.6 (approximately 1 × 10^6^ CFU). After inoculation, the plates were incubated at 37°C for 14 h, and the diameter of growth was measured using a digital caliper (Starrett^®^ 799). Three independent experiments were performed with five replicates of each strain tested, and the data of the three experiments were plotted together in the same graph.

### Macrophage Infection

Macrophage infection assays were performed as previously described (Elsinghorst, 1994) with some modifications. In brief, the murine macrophage-like cell line J774A.1 was cultured in antibiotic-antimycotic-free RPMI 1640 medium (Corning^TM^), containing 10% fetal bovine serum (FBS). A total of 2 × 10^5^ cells/mL were added to 24-well plates (Nunc, Naperville, IL, United States) and incubated at 37°C for 24 h in a humidified atmosphere with 5% CO_2_ for adhesion. Non-adherent cells were removed by washing the wells three times with RPMI 1640. Wild-type SEnPT4 and the mutants were prepared by re-suspending cells from overnight LB plates in RPMI 1640, to obtain OD_600_ of 0.6, diluted 1:100 to obtain a suspension of approximately 2 × 10^6^ CFU/mL, then added to the cells at a multiplicity of infection of 10:1 and re-incubated for 1 h. The cells were then washed twice with PBS to remove non-adherent/invasive cells and re-incubated in a culture medium containing gentamicin (100 μg/mL) for 1 h to eradicate any remaining extracellular bacterial cells. The cells were washed again with PBS. One plate was immediately lysed in 0.5% Triton X-100 and plated for colony counting on LB plates (i.e., 2 h after infection). The other plate was again incubated in RPMI 1640 medium supplemented with 10% FBS and gentamicin (25 μg/mL) for an additional 3 h (i.e., 5 h after infection), which was followed by lysis in 0.5% Triton X-100 and plating for colony counting on LB plates. We performed two independent experiments, with five repetitions of each strain, and the results were plotted together in the same graph.

### Lethal Dose Determination

Female BALB/c mice aged between 6 and 8 weeks were used in the experiments. The mice were housed under specific pathogen-free conditions in the Animal Research Facilities of the State University of Campinas.

Groups of mice were intragastrically inoculated with different doses (10^4^ and 10^6^ CFU) of the wild-type and mutant strains, suspended in 100 μL of PBS, using a gavage needle. Five hours before the inoculation, the food was removed from mice to prevent stomach acidification. The animals were monitored for survival throughout the experiment (30 days after inoculation). The double mutant strain PT4Δ*hupAB* was also tested at a dose of 2 × 10^9^ CFU. This study was carried out in accordance with National Council for the Control of Animal Experimentation (CONCEA) guidelines. All of the animal studies were performed under the Ethics Committee on Animal Research of the University of Campinas approved all the experiments (CEUA: protocols 1920-1 and 4373-1).

### Estimation of PT4Δ*hupAB* Colonization in Mouse Organs

A group of 15 mice was intragastrically inoculated with 100 μL of PBS suspension containing 2 × 10^9^ CFU of PT4Δ*hupAB* using a gavage needle. Another group of 15 mice was inoculated with 3.5 × 10^5^ SEnPT4. The control group consisted of three animals inoculated with sterile PBS, the same solution that was used to prepare the bacterial suspension. On days 3, 7, and 14, a group of 11 mice (5 mice inoculated with PT4Δ*hupAB*, 5 inoculated with SEnPT4, and 1 control) was sacrificed by cervical dislocation, which was followed by the removal of Peyer’s patches and spleen and liver portions. Tissues were weighed and then shredded in a tissue homogenizer (Omni Mixer Homogenizer, Vernon Hills, IL, United States) in 1 mL of PBS. The mixed organ suspension was serially diluted to 10^-2^, and aliquots of 100 μL of the dilutions were plated onto MacConkey’s agar. The plates were incubated at 37°C for 24 h to count the bacteria. After counting, a few colonies were randomly chosen to confirm the presence of mutation by PCR.

### Survival of Immunized Mice Following Challenge With Wild-Type *S. enterica* Strain

Three groups of nine mice were intragastrically immunized by a gavage needle. One group received just one dose of approximately 2 × 10^9^ CFU of PT4Δ*hupAB* on day 0, the second group received the same dose (2 × 10^9^ CFU) of PT4Δ*hupAB* on days 0 and 21, and the third group received two doses of PBS on days 0 and 21. The three groups were challenged with a lethal dose (1 × 10^7^ CFU) of the wild-type SEnPT4 strain 42 days after the first immunization. All mice were monitored for survival through 30 days.

### Detection of Specific Antibodies and Cytokines

Serum and fecal samples were obtained from seven mice per group on days 0, 14, 31, and 49 after the first immunization and assessed for the presence of IgG and IgA, respectively, by ELISA. Briefly, 96-well microtiter plates were coated with 1 μg/well of total *S. enterica* surface-sonicated extract diluted in 0.2 M carbonate buffer (pH 9.6) and incubated overnight at 4°C. Individual samples were added at a dilution of 1:20 in PBS-1% gelatin, and the plates were incubated at 37°C for 2 h. To detect IgA, a rabbit anti-mouse IgA antibody (Sigma-Aldrich, St. Louis, MO, United States) was added at 1:500 dilution, which was followed by incubation with a goat anti-rabbit antibody conjugated with horseradish-peroxidase (Sigma-Aldrich, St. Louis, MO, United States) at 1:1000 dilution. Total IgG reaction was performed by using goat anti-mouse IgG conjugated with horseradish-peroxidase (Santa Cruz Biotechnology) diluted at 1:5000. Color was developed with 3,3′,5,5′-tetramethylbenzidine substrate (TMB), prepared according to the manufacturer’s instruction (Pierce Chemical, Co., Rockford, IL, United States), at 37°C for 15 min. The reaction was stopped with 2 M H_2_SO_4_ before readings were recorded at 450 nm using a Microplate Scanning Spectrophotometer (PowerWave X, Bio-Tek Instruments, Inc., Winooski, VT, United States).

Serum samples were also collected on days 0, 14, 31, and 49 after the first immunization for cytokine quantification on an independent experiment. IFN-γ, TNF-α, IL-2, IL-4, IL-6, IL-10, and IL17A levels were measured by mouse Th1/Th2/Th17 Cytokine Bead Array (CBA) assay (BD Pharmingen) according to manufacturer’s instructions and analyzed using the FACSCanto II cytometer (BD Biosciences).

### Statistical Analysis

Statistical analysis was performed by analysis of variance followed by the parametric Tukey–Kramer or Bonferroni tests using the INSTAT software (GraphPad, San Diego, CA, United States). Results were presented as mean and SEM. *P* < 0.05 was considered statistically significant.

## Results

### *S.* Enteritidis HU Double Mutant Has Affected *in vitro* Growth and Motility

**FIGURE 1:**
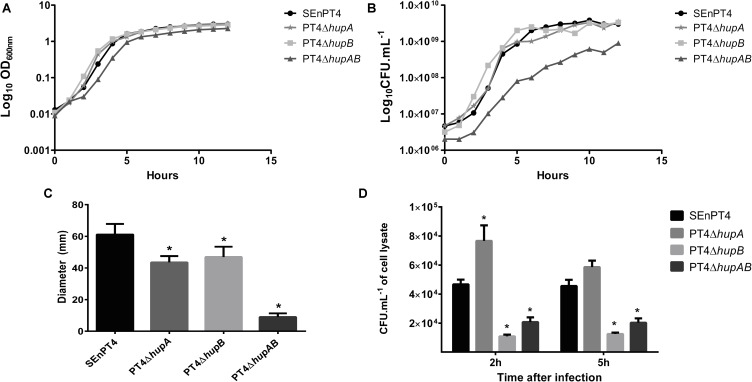
*In vitro* phenotypes of HU-lacking mutants. The growth of all mutant strains and wild-type parental strain SEnPT4 was measured every hour for 12 h by OD_600_ readings **(A)** or CFU counts **(B)**. The growth curves represent the results of three independent experiments. **(C)** The motility capacity of the mutants was also evaluated in 0.35% agar plates, measured after 14 h of incubation. **(D)** J774A.1. macrophages were infected *in vitro* with HU mutants and SEnPT4 parental strains. Graphs show the number of colony forming units (CFUs) of intracellular bacteria recovered at 2 and 5 h after infection. Values of motility and macrophage assay are represented as mean ± SEM of quintuplicate samples of three independent experiments. ^∗^*P* < 0.01 by Tukey’s test as compared to SEnPT4.

Strains lacking HU subunits were compared to the parental strain SEnPT4 in terms of growth. Growth analysis by OD_600_ readings (**Figure 1A**) did not reveal much difference, except for a slight delay in the lag phase of PT4Δ*hupAB*. In contrast, CFU counts showed that the double mutant exhibited affected growth compared to SEnPT4, whereas single mutants exhibited the same growth pattern as the wild-type strain (**Figure 1B**).

Motility analyses demonstrated that HU was important for this phenotype. Both PT4Δ*hupA* and PT4Δ*hupB* mutants displayed less motility in semi-solid agar plates than the parental SEnPT4 strain. The double mutant PT4Δ*hupAB* exhibited sharply reduced motility (**Figure 1C**).

### Macrophage Assay

To evaluate whether deletion of HU affected the ability of SEnPT4 to invade and survive inside host cells, *in vitro* infection of murine J774A.1 macrophages with wild-type and HU mutant strains was performed. At 2 h post-infection, all mutant strains behaved differently from the parental strain. Although the number of PT4Δ*hupA* colonies recovered, at 2 h post-infection, it was higher than that of the parental strain, and the PT4Δ*hupB* and PT4Δ*hupAB* colonies was approximately five and threefold lower than the parental strain, respectively (**Figure 1D**). This pattern persisted for 5 h after infection, except that PT4Δ*hupA* exhibited invasion ability similar to that of the parental strain with no statistical differences (**Figure 1D**). These findings suggest that HU favored invasion and/or survival of *S.* Enteritidis in macrophages and that *hupB* probably plays a more important role during this phase of the infection process.

### HU Double Mutant Is Attenuated and Can Colonize BALB/c Mouse Organs

**FIGURE 2:**
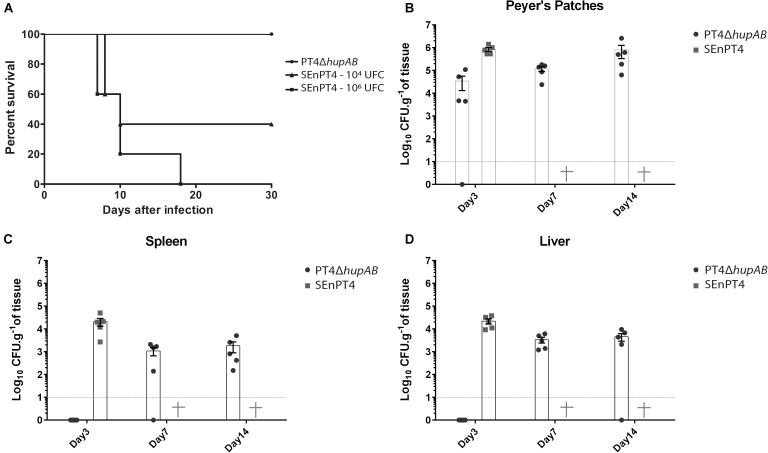
Bacterial cell counting in BALB/c mice organs and attenuation assay following oral inoculation with PT4Δ*hupAB*. **(A)** Mice were orally inoculated with 1 × 10^4^ or 1 × 10^6^ CFU of SEnPT4 to evaluate the virulence of the parental strain; another group was inoculated with 2 × 10^9^ CFU of PT4Δ*hupAB* that exhibited attenuation. Peyer’s patches **(B)**, spleen **(C)**, and liver **(D)** were collected from five animals at 3, 7, or 14 days after inoculation, homogenized in PBS, and plated onto MacConkey’s agar for bacterial counting. Data are representative of two experiments with similar results, and the values are represented by mean ± SD. Some colonies were randomly chosen for PCR confirmation (data not shown).

To investigate how *hupA* and *hupB* deletion influenced the virulence of *S.* Enteritidis, BALB/c mice were inoculated with different doses of PT4Δ*hupAB* and compared with groups inoculated with different doses (1 × 10^4^ and 1 × 10^6^ CFU) of the parental strain SEnPT4 (**Figure 2A**). We tested crescent doses of PT4Δ*hupAB*, including 10^6^, 10^7^, 10^8^, and 10^9^ CFU. In all the doses tested, even at inoculation doses above 1 × 10^9^ CFU of PT4Δ*hupAB*, none of the mice died or showed visible signs of the disease. However, all the animals treated with 1 × 10^6^ CFU of the wild-type strain perished, demonstrating that the PT4Δ*hupAB* mutant is highly attenuated. In preliminary tests PT4Δ*hupA* and PT4Δ*hupB* showed to be as virulent as the wild-type SEnPT4 (data not shown), so they were not tested as a vaccine candidate.

The dynamics of SEnPT4 and PT4Δ*hupAB* infection were analyzed by quantifying the presence of these strains in key organs 3, 7, and 14 days post-infection. **Figure 2** shows that 3 days post-infection, the presence of the mutant was still restricted to the Peyer’s patches, while the wild strain was detected in high numbers at Peyer’s patches (**Figure 2B**), spleen (**Figure 2C**), and liver (**Figure 2D**). However, it was possible to detect the mutant in quantities above 10^3^ CFU/g in the liver or spleen from day 7 to later stages of infection, and while all the mice inoculated with wild strain perished before day 7, no signs of disease were observed in those mice inoculated with PT4Δ*hupAB*. Thus, although PT4Δ*hupAB* is highly attenuated, it could still colonize the host tissues.

### Protection, Antibody Response, and Cytokine Detection After Immunization

**FIGURE 3:**
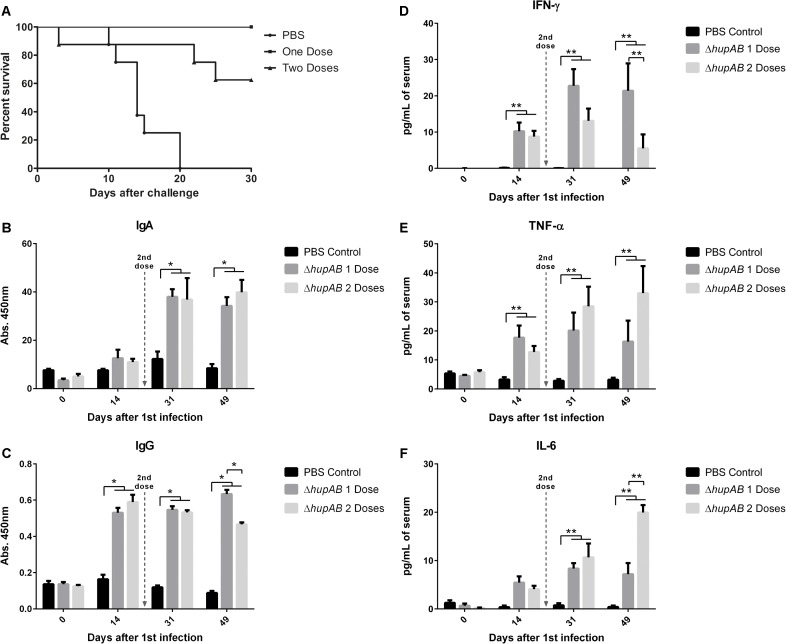
Protection assay, antibody production, and cytokine detection after immunization. BALB/c mice were orally immunized with one (on day 0) or two (on days 0 and 21) doses of 2 × 10^9^ CFU of PT4Δ*hupAB* or inoculated with PBS (control). **(A)** Mice were orally challenged with 1 × 10^7^ CFU of the wild-type strain SEnPT4 21 days after the last immunization. The graph shows the percentage of immunized animals that survived the challenge. Fecal and serum samples were obtained on days 0, 14, 31, and 49 after the first immunization to measure specific IgA **(B)** and total IgG **(C)** by ELISA, respectively. SEnPT4 antigens were used as the coating antigen (1 μg/mL). Results are expressed as the mean ± SEM of OD 450 nm values of seven mice per group and are a representative experiment of two assays. ^∗^*P* < 0.01 compared to the control group with Bonferroni’s test. Cytokine detection in serum collected from groups of nine BALB/c mice after immunization with one (on day 0) or two (on days 0 and 21) doses of 2 × 10^9^ CFU of PT4Δ*hupAB* or with PBS (control). Serum samples were obtained on days 0, 14, 31, and 49 after the first immunization, and levels of IFN-γ **(D)**, TNF-α **(E)** and IL-6 **(F)** were measured by Cytokine Bead Array. Results are expressed as the mean ± SEM of serum concentration (pg/mL). ^∗∗^*P* < 0.05 compared to the control group with Tukey’s test.

To assess the ability of PT4Δ*hupAB* to induce protection, BALB/c mice were inoculated with one or two bacterial doses containing up to 2 × 10^9^ CFU of the mutant strain (**Figure 3A**). A group of nine mice inoculated with only one dose presented 100% survival after challenge with 1 × 10^7^ CFU of wild-type SEnPT4, which is 10-fold higher than the amount required to kill all BALB/c mice. In the same experiment, the group treated with a second dose presented approximately 60% survival, suggesting that the single dose treatment is more appropriate for protection using this vaccination regime. In fact, immunization with one or two doses with 2 × 10^9^ CFU/mL of PT4Δ*hupAB* induced an effective humoral response (**Figures 3B,C**). For the group treated with two doses, however, the total IgG titer clearly decreased after the second dose, which potentially explains the deaths observed in the group of animals immunized with two doses after the challenge.

The same immunization protocol was used to assess serum cytokines on days 0, 14, 31, and 49 after the first dose using a pre-designed CBA assay to analyze IFN-γ, TNF-α, IL-2, IL-4, IL-6, IL-10, and IL17A levels. The levels of IL-2, IL-4, IL-10, and IL17A were undetectable at all the time points assessed (data not shown). However, the levels of IFN-γ, TNF-α, and IL-6 increased on days following immunization (**Figures 3D–F**).

The levels of Th1-related cytokines, IFN-γ and TNF-α, increased 14 days after the first immunization and were continuously higher than those of the control group up to 49 days later (**Figures 3D,E**). After the second dose, however, the level of IFN-γ decreased considerably, which accounts for the deaths observed in the group of animals treated with two doses after challenging. Similarly, IL-6 levels were significantly higher than the control group only 31 days after the first immunization. However, a significant increase was observed in the group that received a second dose 49 days after the first immunization (**Figure 3F**). This result may indicate an increase in the inflammation process, which also could explain the deaths observed after the challenge.

## Discussion

Description of new targets to attenuate *S. enterica* virulence is crucial because invasive non-typhoidal salmonellosis is epidemiologically important and the discovery of new vaccine candidates is urgent (Crump et al., 2015; Tennant and Levine, 2015). Here, we explored a *S*. Enteritidis *hupA* and *hupB* double mutant as a live vaccine candidate that was highly attenuated and elicited a protective immune response.

HU protein, used here as a deletion target to achieve attenuation, is a dimeric NAP that can occur as a homodimer (HUα_2_ and HUβ_2_) or heterodimer (HUαβ) depending on the bacterial growth phase (Claret and Rouviere-Yaniv, 1997). HU of *S*. Typhimurium plays a wide-ranging role in controlling expression of genes involved in adaptation to stress, changes in growth phase, motility, and virulence (Mangan et al., 2011). Our findings regarding the serovar Enteritidis seems to follow a similar pattern, but with some specific differences.

Growth analysis revealed that the single mutants PT4Δ*hupA* and PT4Δ*hupB* did not differ from the parental strain, whereas PT4Δ*hupAB* exhibited significantly decreased growth rate, as evidenced by quantification of viable cells (**Figure 1B**). Furthermore, the motility capacity of all the tested mutant strains was affected in semi-solid agar plates, although the double mutant was the most impacted (**Figure 1C**). These results corroborate with the findings described previously (Mangan et al., 2011) and may be due to the fact that HU coordinates the genomic structure and function (Berger et al., 2010). In contrast with other NAPs, like Fis and H-NS, HU seems to play a mechanistically more direct role in organizing the active transcription units more than regulating it, allowing for independent expression of physically linked genes (Berger et al., 2016).

Furthermore, our data indicate that *hupB* has a major role during macrophage infection compared to *hupA* in *S*. Enteritidis (**Figure 1D**). These data are in contrast with previous results (Mangan et al., 2011) for *S*. Typhimurium. The mechanism by which HU influences the expression of sets of genes is not fully understood (Dillon and Dorman, 2010). However, considering the peculiarities for each region of the genomic structure (Kar et al., 2005) and the role of HU in the regulation of the spatial distribution of RNA polymerase in the nucleoid (Berger et al., 2010, 2016), it is plausible that different HU conformations influence different sets of genes (Mangan et al., 2011). HU–DNA interactions seem to be non-specific; therefore, we hypothesized that the differences in the genomic architecture between the serovars Typhimurium and Enteritidis may be significant for the different phenotypes. Differences among the genetic backgrounds of strains may also be linked to these results; a recently published genomic sequence of *S.* Enteritidis PT4 (Milanez et al., 2016) can be used to elucidate these differences. Thus, further studies are necessary to clarify these differences.

A strain being explored as a live-attenuated vaccine must be sufficiently attenuated (Galen and Curtiss, 2014). Thus, we used BALB/c mice to verify (i) the level of attenuation in the mutants of HU genes and (ii) how the mutant strain can colonize host organs. To the best of our knowledge, no study has determined attenuation caused by deletion of HU genes in *S. enterica* in a murine model, although a previous study has described that *hupA* deletion in *S*. Typhimurium causes attenuation in newly hatched chicks (Turner et al., 1998). Clearly, the double mutant PT4Δ*hupAB* was highly attenuated (**Figure 2A**). However, the same could not be concluded for the single mutant strains, which presented high virulence in preliminary tests even at doses lower than 10^6^ CFU (data not shown).

Although the reasons for the different attenuation characteristics observed in the single-mutants were not further investigated in this study, the less pronounced reduction of some factors, such as motility in semi-solid agar and growth capacity compared to the double-mutant, may provide a basis for future studies. It is likely that the absence of only one of the *hup* genes is being suppressed by the expression of the remaining *hup* gene, similar to that occurring in mutants for the H-NS gene, in which its absence is compensated by increasing expression of StpA, partially reverting the attenuation phenotype (Ali et al., 2014). HU total absence can also be partially compensated by transforming *E. coli* with C-terminal domain of GyrA from *Borrelia burgdorferi* (Ruthenburg et al., 2005). So, to explore HU mutants as vaccine strain, more investigation will be needed to exclude the possibility of phenotype reversion, as previously observed for H-NS (Ali et al., 2014).

Considering the attenuation profile, we tested only the double mutant as the potential vaccine. The colonization pattern demonstrated by attenuated mutants of *S. enterica* varies widely, depending on the degree of attenuation caused by the mutation, but also on the number of bacteria present in the inoculum. *S.* Typhimurium Δ*hfq* mutants, for example, was completely cleared by seventh day post infection with 10^7^ CFU (Allam et al., 2011), whereas mutants Δ*cya*Δ*crp* can be detected in amounts close to 10^3^ CFU/g of liver and spleen when inoculated with 10^9^ CFU of the mutant, even 20 days post infection (Oliveira et al., 2007). In both cases the mutant strains were safe and protective in mice models and could colonize the internal organs in the first days after infection. In fact, a vaccine strain must colonize the host tissues to induce a good immune response (Dunstan et al., 1998; Mastroeni et al., 2001). We observed that PT4Δ*hupAB* infected and colonized key organs in the first 14 days post-infection (**Figures 2B–D**) upon administration of a high number of bacterial cells (2 × 10^9^ CFU). PT4Δ*hupAB* showed spleen and liver colonization only on day 7 after infection, indicating that the first steps of the infection were more affected by the double-mutation. The high level of attenuation and low colonizing ability in the beginning of the infection was partially due to the difficulty in the invasion of macrophages by this mutant and because of growth and motility deficits (**Figure 1**). Nevertheless, approximately 10^9^ cells of this mutant elicited an immunological response that was able to protect 100% of the animals immunized with a single dose (**Figure 3A**).

A balanced cellular and antibody response is also important to establish a good vaccination protocol. It is generally accepted that macrophage activation by cytokines, such as IFN-γ and TNF-α, is important for eradication of *Salmonella* in experimental infection (Stoycheva and Murdjeva, 2005) as these cytokines stimulate NK cells and macrophage effector mechanisms and later ensure T-cell response for pathogen clearance (de Jong et al., 2012). In the same way, antibody production against *Salmonella* plays a notable role in resistance, indicating that both T- and B-cell-mediated immunity is involved in response to *Salmonella* infection (Raupach and Kaufmann, 2001). Considering this, we observed that in the strategy presented here, immunization with only one dose was able to protect 100% of the animals, indicating that both innate and adaptive immune responses may have been elicited.

In our results, protection was preceded by a significant increase in specific IgA and IgG levels along with the increase in the serum levels of IFN-γ and pro-inflammatory cytokines (TNF-α and IL-6). IgA production is important as the first line of defense in intestinal infection. Moreover, Erova et al. (2016) showed, using a different vaccination strategy, that the efficiency of IgA against salmonellosis is associated with its sustained production in animal models following vaccination, demonstrating that it also participates in adaptive immunity. We observed a significant increase in IFN-γ levels in animals receiving only one dose of attenuated PT4Δ*hupAB*, and this profile was sustained at 49 days later after infection. IFN-γ is produced by NK cells and T cells and is important for restricting bacterial replication and macrophage activation, particularly during early stages of infection (de Jong et al., 2012; Kupz et al., 2014).

The production of TNF-α is also important for *Salmonella* clearance from the host cells; TNF-α acts synergistically with IFN-γ to induce the production of nitric oxide (Raupach and Kaufmann, 2001). Both TNF-α and IL-6 production demonstrated in our study may be associated with dendritic cell response, which is related to Th1 cell differentiation during T-cell receptor (TCR) stimulation (Arango Duque and Descoteaux, 2014; Das et al., 2017). However, secretion of high levels of TNF-α may result in immunopathology and increase in mortality, possibly due to lipopolysaccharide (LPS)-mediated septic shock (Raupach and Kaufmann, 2001).

Our results showed an increase in mortality in the mice group that received a second dose of the vaccine and presented high levels of TNF-α 49 days post-infection and, consequently, decreased protection (approximately 60%) against the wild-type strain. The second dose resulted in a decrease in serum levels of IgG and IFN-γ and higher IL-6 levels at 49 days after the first immunization; the reason for this is unclear, but it can be speculated that the second dose with more than 10^9^ CFU may have led to hyperstimulation of the immune system because of the high amount of LPS, thereby inducing a strong inflammatory response.

Therefore, future studies must focus only on single-dose treatment because the protection observed with a single dose is a desirable characteristic for vaccines. Overall, our data indicate that the strain was able to induce a balanced immune response at single dose, involving innate and adaptive immune mechanisms, suggesting that *hupAB* mutants should be further explored for the development of potential live-attenuated vaccine for salmonellosis. More tests will still be needed to determine if the immunological response can induce a long-lasting protection and also if this protection can be extended to other *Salmonella* serovars. In future experiments, the resistance cassette should also be removed, since the strain to be explored as vaccine couldn’t hold it during field tests.

The results presented in this study allow us to state that *hupAB* mutants could potentially be used as a live-attenuated vaccine. Moreover, we observed that this HU-lacking mutant is highly attenuated and is, therefore, safe for use as a live vaccine in addition to being able to elicit an immune response that is capable of protecting mice against a challenge with wild-type *S. enterica* Enteritidis PT4. However, further studies are still needed to better understand the immune response generated by this strain.

## Author Contributions

GM and MB designed and executed the experiments, performed the analyses of the results, and wrote the manuscript. GM, CW, and RR handled the animal experiments. GM and LT established and performed the macrophage infection assay. MA performed Cytokine Bead Array. MR-B and AO designed the ELISA experiment for antibody quantification. All authors were major contributors in writing the manuscript and have read and approved the final manuscript.

## Conflict of Interest Statement

The authors declare that the research was conducted in the absence of any commercial or financial relationships that could be construed as a potential conflict of interest.
